# Supplementary material for: Chromothripsis during telomere crisis is independent of NHEJ, and consistent with a replicative origin
Source: Genome Res. 2019 May;29(5):737–49. doi: 10.1101/gr.240705.118 (PMC6499312; doi:10.1101/gr.240705.118)
Supplement: Supplemental Material [file supp_gr.240705.118_Supplemental_file_1.zip › contigs/annotated_contigs/DB78/contig.2.DB78_length_632_mean_cov_15.664556962.docx]

**DB78_length_632_mean_cov_15.664556962**

```
AAGCCTCTCTAATTGGGAAATTACACGCAAAAACCCAGAAAAATTGTATCCCTCCAAAAGGTTTCATAGGCACTCAGAATCTCTAGCCA
 >chrX:115336443-115336684 + E=3e-133
GGTGATTGGTGAAGATATTCTCCTGTATTAAGCCAGTCTGTAGATACCAAAAGATGATTTTTTAACAAATGCACAAAATTCAGGAAAAA

ATTATAAGGCACATTAAAAATGGAAGTATGCCTCAGGGAAAGGAAACAATAAATCACTAGATA|CACTAG|ATACACTGTTCGTGGGAA
                                                                      >chrX:115338645-1
TGTAAATTAGTACCACCTCTAGGGAAAACAATATGGAGACTTCTCAAAGAACAAAAAGTAGATCTACATTCAAACAAGCAATCCCACTA
15339030 + E=5e-220
CTTCATATATGCCCAAAGAAAAAGAAGTCATATCACAAAGACACCAGCATTTATATGTTTATCACAGTACAATTTACAATTGCAAAGAT

ATAGAATCAACCTCAGTGCCCATCAACTGATAAGTGAATAAAGAAAATGTATATACACCATGAAGTATTACTCAGCCAGAAAAAAAGAG

TGAAACAGTGTCTTTTGCAGCAATTTGGATAGAACTGGAGGCCTTTATTCTAAGTGAAGTAACTCAGAAATGGAAAATCAAATTCTGCA

TGTTCTCACTT
```
